# Is it possible to identify physical-motor profiles of preschool children on their association with selected biosocial factors?

**DOI:** 10.3389/fpsyg.2024.1302402

**Published:** 2024-02-14

**Authors:** Pedro Gil-Madrona, Luisa Losada-Puente, Paula Mendiri, César Sá, Inês P. Silva, Linda Saraiva

**Affiliations:** ^1^Department of Didactics on Physical, Artistic and Music Education, Faculty of Education, Albacete, University of Castilla La Mancha, Ciudad Real, Spain; ^2^Department of Specific Didactics and Methods of Research and Diagnosis in Education, Faculty of Educational Sciences, University of Coruña, A Coruña, Spain; ^3^Escola Superior de Educação de Viana do Castelo, Instituto Politécnico de Viana do Castelo, Viana do Castelo, Portugal; ^4^Center for Research and Innovation in Education, Porto, Portugal

**Keywords:** physical-motor development, preschool children, age, sex, prematurity, body mass index, extracurricular physical activities

## Abstract

Biosocial factors play a crucial role in the physical-motor development (PMD) of children during the preschool age. The present study aims to identify physical-motor profiles throughout preschool age (3–6 years) and explore associations between profiles and selected biosocial factors such as age, sex, prematurity, weight, height, BMI, and participation in extracurricular physical activities. Data from 412 typically developing children (46.6% girls and 53.4% boys), aged 35–71 months (M = 51.21, SD = 10.47) was collected using the Psychomotor Activities Checklist and specifically the scale of Psycho-Motor Aspects. Cluster analysis made it possible to define four different childhood PMD profiles. High PMD; High PMD except left laterality; medium-low PMD; and low PMD. High PMD profile includes older children, with anthropometric measurements closer to the WHO recommendations, fewer preterm children, and greater participation in extracurricular physical activities. Low PMD profile includes younger children, with weight slightly above and height slightly below the WHO recommendations and low participation in extracurricular physical activities. This study allows us to identify specific trends that may be decisive for the motor development of children throughout preschool age, highlighting selected biological variables and participation in extracurricular physical activities.

## 1 Introduction

All healthy children have the potential to develop and learn motor skills during early childhood. Nevertheless, research shows that children may have different developmental pathways to achieve motor proficiency (Hadders-Algra, 2018; Boonzaaijer et al., 2021). Understanding and explaining this inter-individual variability is still challenging in contemporary research.

In the past, the theoretical and empirical approach to this topic has been portrayed by the “nature versus nurture” dichotomy, suggesting that genetics (nature) or environmental factors (nurture) were the primary drivers of motor development. Researchers abandoned this approach and recognized that acquiring a broad motor repertoire during early childhood is determined by the complex interaction between biological factors and environmental conditions in which a child lives and grows (Krebs, 2009; Gabbard, 2014). Among various contemporary approaches, Bronfenbrenner’s Bioecological Theory (Bronfenbrenner, 2005) provides a useful theoretical and conceptual framework for better understanding the biosocial influences on children’s motor development.

In the last decade, several reviews and systematic reviews support that a wide range of biosocial factors influences children’s motor development (Lubans et al., 2010; Barnett et al., 2012; Holfelder and Schott, 2014; Livonen and Sääkslahti, 2014), that can contribute to the variability observed in children’s acquisition and refinement of motor skills. Heterochrony in children’s motor development can be explained by the rate of growth and maturation (Drenowatz and Greier, 2019; Werneck et al., 2020; Pereira et al., 2021). For instance, growth hormone has been found to impact the central nervous system, including the brain. It plays a role in developing neural structures and may influence cognitive and motor functions. Variations in the timing of hormonal changes during childhood can contribute to differences in cognitive and motor development among children (Webb et al., 2012).

Variations in children’s motor development can also be explained by age, gender, physical activity, and preschool-based programs (Livonen and Sääkslahti, 2014). The systematic review by Barnett et al. (2012) on correlates of motor competence in typical development children and young people (3–18 years), using an ecological approach, reinforces that increasing age was the most consistent correlate of all aspects of motor competence. The weight status (healthy), sex (male), and socioeconomic background (higher) were consistent correlates for specific motor skills. In the same study, the authors also emphasize the positive correlation between physical activity and composite and motor coordination skills, despite the relationship that is not consistent for object control and locomotion skills.

Regarding the age effect, research showed that age was positively associated with locomotor, object control, and stability skills (Barnett et al., 2012; Valentini et al., 2016; Bolger et al., 2018; Li et al., 2023); however, the magnitude of its effect tends to decrease or even disappear with age, given that children reach the expected motor proficiency in some motor skills. On this issue, it is essential to point out that age reflects the child’s biological and neurological maturity and the accumulated effects of stimulation and environmental factors. It is also reasonable that the interaction of maturation and environmental effects may help to explain the increased variability in motor development with advancing age.

The relationship between sex and motor skills in preschool children has been studied (e.g., Saraiva et al., 2013; Valentini et al., 2016; Navarro-Patón et al., 2021). For instance, Saraiva et al. (2013) concluded that in preschool age, there were significant differences between boys’ and girls’ performance in grasping, visual-motor integration, and object manipulation subtests. Girls had superior performance in grasping and visual-motor integration skills, while boys performed better in object manipulation skills. This different motor profile between boys and girls is consistent with most studies found in the literature (Saraiva et al., 2013; Valentini et al., 2016; Navarro-Patón et al., 2021; Escolano-Pérez et al., 2022), and it has been attributed to environmental and educational influences but also to biological factors such as advanced neurological development favoring girls, and to some morphological characteristics favoring boys (Ikeda and Aoyagi, 2008; Gabbard, 2011). However, it is essential to note that the magnitude of the sex effect varies according to age and motor skill specificity. In the study by Saraiva et al. (2013), it was found that boys’ advantage in object manipulation skills becomes progressively greater throughout preschool age, which suggests a progressive modulation effect of motor experiences across childhood. These differences between boys and girls in motor skills at preschool age can be explained by support and opportunities for structured and unstructured physical activity at home, school, and the community. On this issue, Valentini et al. (2016) warn that opportunities for practicing and learning motor skills must be structured and developmentally appropriate for the children, providing an adequate challenge to improve motor proficiency. Previous systematic reviews have shown that motor intervention programs implemented throughout childhood are crucial to improving motor competence and increasing participation in physical activity (Hardy et al., 2014; Holfelder and Schott, 2014; Wick et al., 2017; Coppens et al., 2021; Jaakkola et al., 2021).

Researchers have also examined the relationship between motor competence and potential health benefits in children and adolescents (Lubans et al., 2010; Jones et al., 2020; Xin et al., 2020). From this investigation emerges the idea that motor competence is positively associated with physical activity and is inversely correlated with weight and BMI status. However, the strength of these relationships seems to vary across age and specificity of motor skills. For example, overweight and obese children tend to have poorer performance than their non-overweight peers, and these differences appear to be more apparent for locomotor skills than object manipulation skills (Okely et al., 2004; Cliff et al., 2012). Likewise, underweight children tend to have less motor coordination and muscular power than normal-weight children, but underweight and normal-weight youth generally perform better than overweight or obese youth (Lopes et al., 2018; Verbecque et al., 2022).

Finally, it is also important to mention that distal biological factors such as gestational age and birth weight can also explain motor variability. Lower birth weight and gestational age are strongly related to poorer motor outcomes in the first years of development (e.g., Oudgenoeg-Paz et al., 2017). Studies have shown that premature birth (birth before 37 weeks of gestation) can have various effects on a child’s development, including motor skills, muscle strength, and physical activity levels than term-born children (FitzGerald et al., 2021; Karageorgi et al., 2021). This adverse effect of preterm birth on a child’s motor performance may persist into adulthood (Moreira et al., 2014; Allotey et al., 2018), depending on the extent of prematurity and associated complications.

Based on the assumption that the influence of biosocial factors on motor proficiency throughout preschool age varies according to age and motor specificity, the present study sought to identify physical-motor profiles throughout preschool age (3–6 years) and explore associations between profiles and selected biosocial factors (age, sex, prematurity, weight, height, BMI, and participation in extracurricular physical activities).

## 2 Materials and methods

### 2.1 Sample

A convenience sample of 412 typically developing children aged between 35 and 71 months (M = 51.21, SD = 10.47) was recruited from 19 children’s classrooms from different kindergartens in the province of Albacete. Children included in the study met the following criteria: age between 36 and 71 months, absence of any known intellectual, physical, or emotional disabilities, as well as without special educational needs, as proven by records of special education teams. The sample has been non-probabilistic, the sampling technique has been used on purpose, as voluntary collaboration has been requested in all schools in Albacete province, and we have finally worked with the schools that agreed to participate.

In total, 46.60% of the sample were girls, and 53.40% were boys. The average weight ranged from 10.90 to 28 kg (M = 17.10, SD = 2.63), and height ranged from 82 to 126 cm (M = 103.72, SD = 7.46). The body mass index (BMI) ranged from 12.18 to 21.43 (M = 15.86, SD = 1.43). Most children were born at full term (i.e., not premature: *n* = 323, 78.4%). There were 67 missing values. Only 21.8% of the children played extracurricular physical activities (*n* = 90), mainly football, and to a lesser extent, swimming, tennis or paddle, martial arts, rolling, or dancing, versus a majority who did not practice (*n* = 322, 78.16%).

The parents or legal guardians of the preschool children were informed about testing procedures, the guarantee of anonymity and confidentiality of data, and corresponding written consent was obtained.

### 2.2 Measures

#### 2.2.1 Checklist of psychomotor activities-psychomotor aspects scale

In this study, the Psychomotor Activities Checklist (CPA; Romero Martínez et al., 2018) was used to measure the physical-motor development of children, and specifically the scale of Psycho-Motor Aspects (PSAS), composed of five dimensions: Laterality (LAT, six items), Dynamic Coordination (DC, six items), Tonic-Postural Control (CTP, three items), Motor Execution (ME, three items), and Balance (BAL, five items). LAT is the dominance of one side of the brain in controlling activities or functions, measured by tasks such as “hitting objects with the left leg.” DC is the ability to apply skills, knowledge, and situational awareness, measured by tasks such as “jumping with one foot.” ME is composed of overt and volitional movement, for example, “lies down with his back straight.” BAL is a state of equilibrium or equipoise, measured with actions such as “maintains balance by walking on a curve.”

The tasks in each motor domain can be found in Table 1 alongside the reliability values, contrasting the original scale with that of the present study. It should be noted that in the present study, the dimension of laterality was explored in two sub-dimensions, given that the literature points out that its establishment, including handedness, typically occurs during early childhood, around the age of 3 to 5 years (Scharoun and Bryden, 2014; Bondi et al., 2020).

**Table 1:** Tasks evaluated and Cronbach’s alfa (95% confidence interval) for each motor domain of the PSAS scale.

Dimension	Item/task	α (original)	α (present study)
Laterality (LAT)	Grasps objects with the left hand. Grasps objects with the right hand. Hits objects with the left leg. Hits objects with the right leg. Throws objects with the left hand. Throws objects with the right hand.	0.57 (0.56–0.60)	Original LAT = 0.83 (0.81–0.82) Right LAT = 0.88 (0.89–0.80) Left LAT = 0.91 (0.84–0.92)
Dynamic Coordination	Is able to roll on a surface. Jumps with both feet together. Jumps with one foot. Is able to move sideways. Is able to walk backward. Runs freely without difficulty.	0.77 (0.75–0.81)	0.89 (0.85–0.90)
Balance	Maintains balance by walking in a straight line. Maintains balance by walking along a curved line. Maintains balance by walking on a trestle. Maintains balance by walking on a bench. Is able to maintain a balanced posture.	0.87 (0.86–0.92)	0.86 (0.77–0.82)
Motor Execution	Is able to use the materials correctly. Is able to jump over obstacles. Is able to circle around obstacles.	0.65 (0.63–0.67)	0.86 (0.78–0.81)
Tonic Postural Control	Moves following the indicated rhythm. Runs and is able to stop at a signal. Lies down flat on the back.	0.60 (0.58–0.63)	0.88 (0.84–0.88)

Furthermore, from the analyses carried out, it could be observed that, although Cronbach’s Alpha values were acceptable when separating the items relating to left and right laterality, an excellent index was obtained in both cases.

Participants were evaluated by their teachers using a 5-point Likert scale from 1 (never) to 5 (always), depending on their ability to perform the proposed task on each item. Further details on the CPS’s psychometric properties (validity and reliability) may be consulted in Romero Martínez et al. (2018).

#### 2.2.2 Anthropometric measures

Statures and weight were measured using standard procedures (Lohman et al., 1988), and the Body mass index was calculated as weight (BMI = kg/m^2^).

#### 2.2.3 Birth and extracurricular physical activities data

A questionnaire was applied to parents to collect the child’s birth date, gestational age, and extracurricular physical activity data. The prematurity definition for this study is considered in the reports mentioned above (children born before week 37).

### 2.3 Procedures

In the first moment, different kindergartens and respective teachers in the province of Albacete, Spain, were contacted. An explanatory document for the research’s purposes was presented to the school’s directors, and the parents were invited to participate. Informed consent was requested from parents who agreed to participate. This moment occurred between December 2022 and February 2023.

In the second moment, teachers were training to use the CPA instrument, more specifically the PSAS, and posteriorly, the teachers evaluated the children with the help of a research team member. This work occurred in children’s natural context, that is, in their schools and usual work classrooms, and in the presence of reference adults with whom they are familiar (teachers, internship students, and researchers). The researchers and teachers observed the children at three moments of the school day: in the classroom, in the gym, and during recess. The parents were asked to answer a questionnaire about their children’s birth date, gestational age, and extracurricular physical activity.

The third moment, the last, was dedicated to joining all data and realizing all the analysis.

This research was first submitted for evaluation by the Ethics Committee of the University of Castilla La Mancha, which issued a favorable report. Subsequently, approval was sought and obtained from the families for the children to participate in this study.

### 2.4 Data analysis

Firstly, descriptive statistics (means, standard deviations, skewness, and kurtosis) and Pearson correlations were calculated among all measures. Pearson’s *r*-value varies between [−1 and 1]. Values closer to 1 indicate a higher degree of relationship between variables (David and Sutton, 2004).

Secondly, hierarchical agglomerative cluster analysis was used to identify groupings of students in the data set according to their scores on the right and left laterality, balance coordination, motor performance, and tonicity. Previously, data with missing values on any observation items were removed, and those with missing values only on the socio-demographic data were kept. For carrying out the analyses, a series of steps have been carried out, whereby the cases start as individual clusters and, step by step, the most similar clusters are joined to give rise to a cluster containing all cases until obtaining a cluster containing all cases (Mojena, 1977; Clarworthy et al., 2005). The analyses were performed with Machine Learning in Jasp 0.17.1.0, using neighborhood-based clustering (K-means clustering or generalized Lloyd Algorithm, GLA) method in which the squared Euclidean distance is used to measure dissimilarity between a data point and its cluster representative, considering the cohesion degree the of the neighborhood of an object and the coupling degree between neighborhoods of objects (Cao et al., 2009; Lai and Huang, 2011; Schubert, 2022). This method allows us to reduce the computational complexity of Ward’s method (Lai and Huang, 2011).

We used the stopping rules to determine the final cluster solution (Mojena, 1977; Clarworthy et al., 2005; Baidari and Patil, 2020) by examining the agglomeration schedule and the Elbow criterion (Schubert, 2022). An inconsistent increase in the dissimilarity measure suggests that the groups that came together at that stage were quite different and that the grouping should cease at an earlier stage. To prevent the researchers’ bias associated with the subjectivity of this method, we also incorporate formal rules and equations to determine the number of clusters in a sample (Clarworthy et al., 2005). Therefore, we have relied on a combination of fit indices and methods and the interpretability of the solutions based on theory (Xu and Núñez, 2023). Thus, the pseudo-F statistic (Calinski and Harabasz, 1974, as cited in Baidari and Patil, 2020) has been included. Large values of the Calinski–Harabasz pseudo-'F index indicate distinct clustering, whereas small values indicate less clearly defined cluster structure, and the Silhouette index (Kaufman and Rousseeuw, 1990), a coefficient (range [−1, 1]) that reflects the closeness of a point within the cluster and in the neighboring clusters. We also considered the maximum diameter, the minimum separation, and the Dunn Index (Clarworthy et al., 2005). Compact and well-separated clusters in the data set result from the small diameter of the clusters and the large distance between the clusters; thus, the Dunn index is maximized. A final criterion was to consider adequate and feasible only those profiles that are composed of more than 5% of the sample reflecting enough profile extraction (Xu and Núñez, 2023).

After identifying the optimal number of profiles, additional analyses were conducted to test the validity of the classification. The analysis of variance (ANOVA) test was carried out to test whether there were statistically significant differences among clusters on all measures and to examine whether the clusters differed in age, weight, height, and BMI. The effect size (*η2*) was calculated and interpreted according to (Cohen’s 1988, as cited in Xu and Núñez, 2023), that is η^2^ = 0.01 (*d* = 0.20) a small effect size; η^2^ = 0.059 (*d* = 0.50), a medium effect size; and η^2^ = 0.138 (*d* = 0.80), a large effect size. Tukey’s *post-hoc* test was used for pairwise differentiation. The chi-square test was performed to test the differences related to other biosocial qualitative variables such as sex, prematurity, and participation in extracurricular physical activities. Cramer’s V was calculated as a correction that is applied to the Chi-square coefficient. The closer this index is to 1, the greater the association between variables (David and Sutton, 2004). All values were considered significant for a value of p of less than 0.05 (95% of confidence level).

## 3 Results

### 3.1 Descriptives

Table 2 presents means, standard deviations, skew, kurtosis, and Pearson correlations among all measures. All of them were found to be significantly positively correlated.

**Table 2:** Descriptive statistics and Pearson correlations.

Measure	1	2	3	4	5	M	SD	Skewness	Kurtosis
1. Right laterality						4.26	0.91	−1.23	0.93
2. Left laterality	0.24**	-				3.03	1.12	0.35	−0.79
3. Coordination	0.56**	0.40**	-			3.83	0.93	−0.86	0.47
4. Balance	0.53**	0.44**	0.80**	-		3.86	0.97	−0.80	0.22
5. Motor Execution	0.56**	0.31**	0.65**	0.66**	-	4.38	0.76	−1.49	2.09
6. Tonicity	0.61**	0.42**	0.68**	0.63**	0.72**	3.85	0.95	−0.98	0.21

*n* = 412. **p < 0.001.

### 3.2 Hierarchical clustering

**Figure 1:**
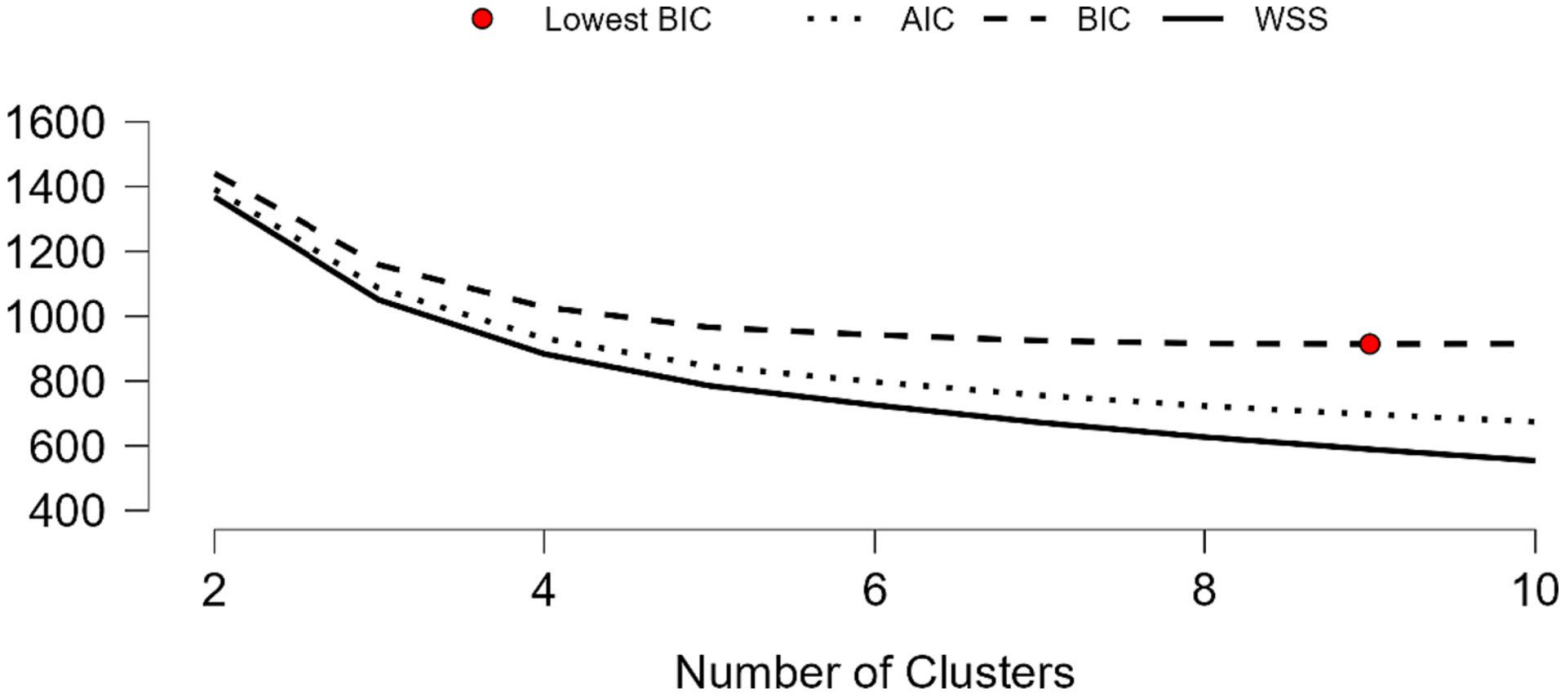
Elbow method plot for 9-clusters solution.

The first agglomerative hierarchical cluster analysis optimized according to the BIC index provided a solution consisting of nine clusters. Figure 1 shows through the Elbow Method Plot the percentage of variance explained by each cluster, gathered in the first four clusters. The change in the inertia value (“elbow”) can be observed in the line represented by WSS (sum of squared errors) that breaks off in cluster 4.

Furthermore, the Silhouette, CHI, and Dunn Index are presented in Table 3. Despite having an acceptable 9-clusters solution, additional refinements were carried out to test the improvements offered by lower clustering, given the aim of extracting a more compact solution. Specifically, an additional test was carried out with five, four, and three clusters (Table 3).

**Table 3:** Number of clusters based on agglomerative hierarchic analysis and fit index.

Clusters	N	R^2^	Silhouette	CHI	Maximum diameter	Minimum separation	Dunn index
9	412	0.76	0.25	160.12	4.75	0.27	0.06
5	412	0.68	0.28	217.88	5.15	0.40	0.08
4	412	0.65	0.28	243.56	5.67	0.40	0.07
3	412	0.57	0.30	275.50	6.66	0.40	0.06

Table 3 shows the index extracted for different solutions. It was observed that R2 decreased as the number of clusters dropped, while the Silhouette Index improved considerably accordingly, reaching acceptable values from 5-clusters solution upwards (>0.25). Similarly, the CHI Index reported more satisfactory results in solutions composed of a smaller number of clusters, thus suggesting a better-defined structure in these latter solutions. As for the Dunn index, the overall results are less satisfactory since the diameter of the clusters increases—it is desirable for them to be more compact—although the separation between them is maintained from five clusters onwards. The lack of improvement in the reduction to three clusters is identified here, so the 4-cluster solution was chosen.

**Figure 2:**
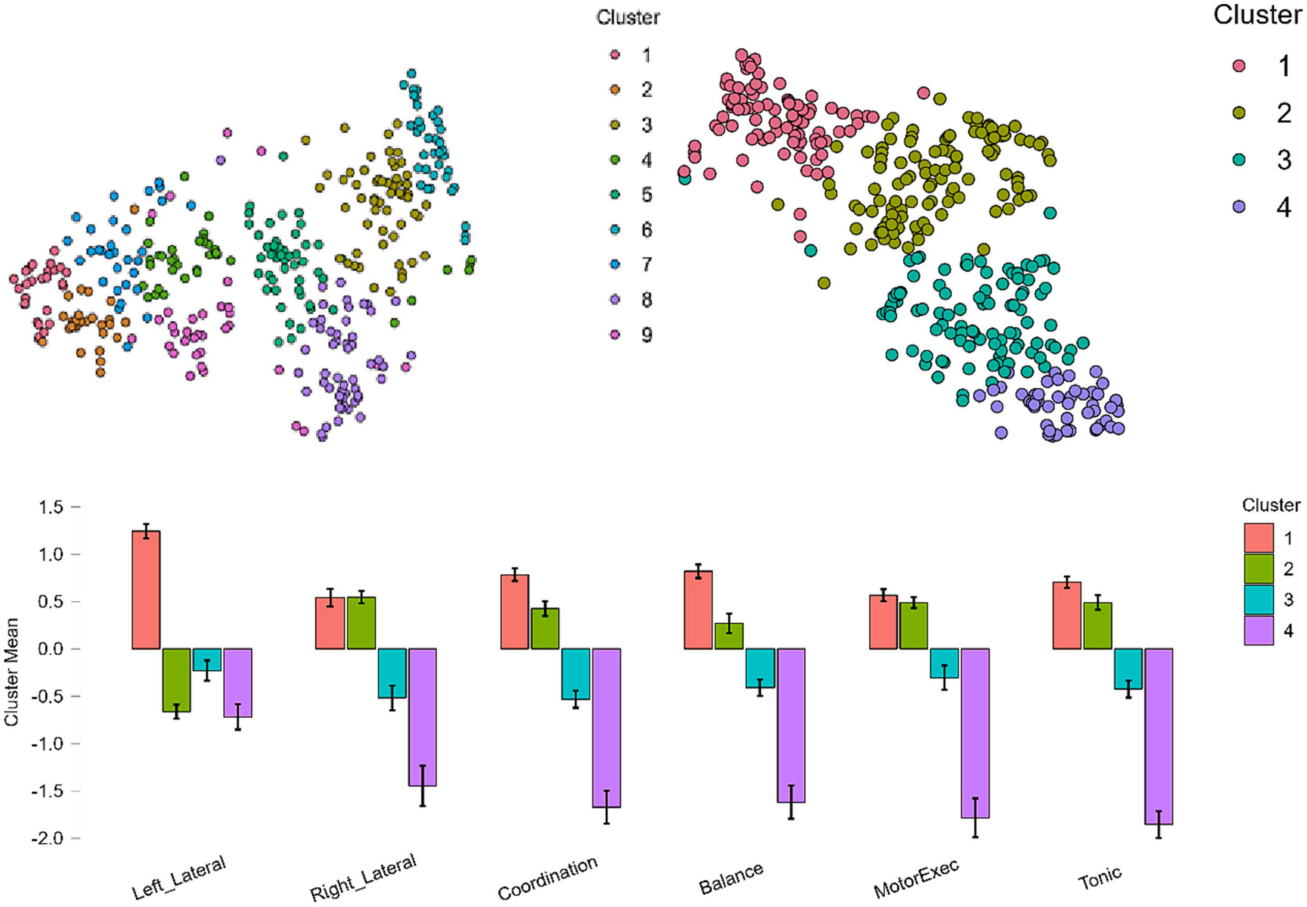
T-SNE cluster plots for 10-cluster and 4-cluster solutions, and cluster mean for 4-cluster solution.

Figure 2 presents two graphs of the t-distributed stochastic neighbor embedding (t-SNE) plots. These plots show the clustering between cases according to their distance in the 2D map. The comparison between the plots for nine clusters (left) and four clusters (right) shows the differences in the island groupings of the datasets. Complementarily, the cluster mean plot allows us to clearly identify the four groupings that are extracted in each cluster: High PMD (cluster 1); High PMD, except left laterality (cluster 2); medium-low PMD (cluster 3); and low PMD (cluster 4).

### 3.3 Profile characterization

Table 4 presents the mean values and deviation of the participant’s scores in each cluster. An analysis of variance is included, showing statistically significant differences between clusters in all the measures used, with a large effect size (η^2^ > 0.14).

**Table 4:** Between-group differences in the dimensions of physical-motor development for each profile.

	Cluster 1 (*n* = 121)		Cluster 2 (*n* = 129)		Cluster 3 (*n =* 107)		Cluster 4 (*n* = 55)		*F*(3, 408)	η^2^
Measure	M	SD	M	SD	M	SD	M	SD		
Right laterality	4.75	0.57	4.76	0.41	3.79	0.75	2.95	0.87	152.48**	0.53
Left laterality	4.41	0.58	2.29	0.57	2.77	0.76	2.22	0.67	282.78**	0.68
Coordination	4.55	0.41	4.22	0.51	3.33	0.53	2.27	0.73	295.13**	0.69
Balance	4.66	0.10	4.12	0.70	2.46	0.53	2.29	0.77	217.27**	0.62
Motor execution	4.81	0.32	4.75	0.30	4.15	0.62	3.02	0.70	221.41**	0.62
Tonicity	4.52	0.38	4.31	0.50	3.45	0.54	2.09	0.61	362.60**	0.73

*p < 0.001. High PMD (Cluster 1); High PMD, except left laterality (Cluster 2); medium-low PMD (Cluster 3); and low PMD (Cluster 4).

*Post-hoc* tests (Table 5) reported the presence of statistically significant differences between all contrast groups in all variables, except between the High PMD group (cluster 1) and High PMD, except left laterality (cluster 2) in right laterality, and between the latter concerning low PMD (cluster 4) in left laterality.

**Table 5:** *Post-hoc* tests for pairwise differences between profiles according to physical-motor development variables.

	Cluster 1–Cluster 2		Cluster 1–Cluster 3		Cluster 1–Cluster 4		Cluster 2–Cluster 3		Cluster 2–Cluster 4		Cluster 3–Cluster 4	
	M diff (SE)	t	M diff (SE)	T	M diff (SE)	t	M diff (SE)	t	M diff (SE)	t	M diff (SE)	t
Right laterality	−0.01 (0.08)	−0.1	0.96 (0.09)	11.6**	1.81 (0.10)	17.7**	0.97 (0.08)	11.8**	1.81 (0.10)	17.9**	0.84 (0.11)	8.1**
Left laterality	2.13 (0.08)	26.3**	1.64 (0.08)	19.4**	2.19 (0.10)	21.1**	−0.48 (0.08)	−5.8**	0.06 (0.10)	0.6	0.55 (0.10)	5.2**
Coordination	0.33 (0.07)	4.9**	1.22 (0.07)	17.6**	2.28 (0.09)	26.8**	0.89 (0.07)	12.9**	1.95 (0.08)	23.1**	1.06 (0.09)	12.2**
Balance	0.53 (0.08)	6.9**	1.2 (0.08)	14.9**	2.37 (0.10)	24.1**	0.66 (0.08)	8.3**	1.84 (1)	18.8**	1.18 (0.10)	11.7**
Motor execution	0.06 (0.06)	1.0**	0.66 (0.06)	10.6**	1.78 (0.08)	23.4**	0.60 (0.06)	9.8**	1.72 (0.08)	22.8**	1.12 (0.08)	14.4**
Tonicity	0.20 (0.06)	3.2*	1.07 (0.07)	16.2**	2.42 (0.08)	30.0**	0.86 (0.07)	13.3**	2.22 (0.08)	27.8**	1.36 (0.08)	16.5**

*p < 0.05, **p < 0.001. High PMD (Cluster 1); High PMD, except left laterality (Cluster 2); medium-low PMD (Cluster 3); and low PMD (Cluster 4).

Description of the four clusters (profiles) regarding biosocial variables are presented in Table 6.

**Table 6:** Description of the four clusters (profiles) regarding biosocial variables.

		Frequency (%)	M	SD	Skewness	Kurtosis	95% CI Mean	
							Upper	Lower
Cluster 1: High PMD (*n* = 121)	*Sex: Male*	63 (52.07)						
	*Age*	121 (100)	57.68	7.50	−0.66	0.34	59.01	56.34
	*Weight*	117 (96.69)	17.44	2.53	1.36	3.09	17.89	16.98
	*Height*		105.78	5.46	0.53	<0.01	102.65	97.07
	*BMI*		15.55	1.49	0.34	1.58	15.82	15.27
	*Prematurity: Yes*	4 (3.39)						
	*Extracurricular physical activities: Yes*	41 (33.88)						
Cluster 2 High PMD, except left laterality (*n* = 129)	*Sex: Male*	60 (46.51)						
	*Age*	129 (100)	50.60	10.16	−0.11	−1.10	46.99	41.66
	*Weight*	105 (81.40)	17.44	2.37	−0.57	1.02	17.89	16.98
	*Height*		104.86	6.80	−1.20	2.33	106.16	103.56
	*BMI*		15.82	1.30	0.71	1.90	16.07	15.57
	*Prematurity: Yes*	5 (5.26)						
	*Extracurricular physical activities: Yes*	26 (20.16)						
Cluster 3 Medium-Low PMD (*n* = 107)	*Sex: Male*	63 (58.88)						
	*Age*	107 (100)	48.18	10.15	0.19	−1.17	50.10	52.35
	*Weight*	75 (70.09)	16.63	2.82	0.79	2.03	17.27	15.99
	*Height*		100.81	8.83	−0.36	−0.75	102.81	98.82
	*BMI*		16.33	1.52	0.99	1.15	16.67	15.99
	*Prematurity: Yes*	7 (7.61)						
	*Extracurricular physical activities: Yes*	19 (17.76)						
Cluster 4 Low PMD (*n* = 55)	*Sex: Male*	34 (61.82)						
	*Age*	55 (100)	44.33	10.07	0.87	−0.46	46.99	41.67
	*Weight*	37 (67.27)	16.03	2.89	1.82	7.31	16.96	15.10
	*Height*		99.86	8.67	0.53	0.04	102.65	97.07
	*BMI*		16.01	1.21	−0.46	0.82	16.40	15.62
	*Prematurity: Yes*	6 (15.00)						
	*Extracurricular physical activities: Yes*	4 (7.27)						

Profile 1 had high values in all dimensions of physical-motor development, which placed it above the rest of the clusters. It comprised an equal number of boys and girls, with the lowest number of cases of prematurity (only 4 cases, 3.39%), of whom more than 30% played extracurricular physical activities. The mean age in this group was 57.7 months (SD = 7.5), and concerning weight and height, in this case, they were approximately in line with WHO standards (see Table 5), although slightly below in terms of height.

In profile 2, high values were found in all dimensions of physical-motor development except for left laterality. It stood out, above all, in right laterality compared to the rest of the groups. The mean age of this group was 50.6 months (SD = 10.16), and five children (5.26%) were preterm. The weight and height of the group were higher than WHO recommendations (Table 5), and participation in extracurricular physical activities was low (79.84% did not participate in extracurricular physical activities).

The group comprising Profile 3 obtained intermediate-low values in most of the analyzed variables. Balance stood out most negatively, followed by left laterality, while motor execution and right laterality did so more positively, together with tonicity. A higher number (almost 20%) of boys versus girls composed this profile, mostly born at term (92.4%), with an average age of 48.18 months (SD = 10.15). The average childhood weight grouped in this profile was slightly higher than the standards proposed by World Health Organization (2006; Table 5), while height was slightly lower. Physical activity in this group was higher than one-fifth of the total number of children.

Profile 4 had the lowest scores on all variables analyzed. This group consisted of more boys than girls (a difference of more than 20%) with a mean age of 44.33 months (SD = 10.07). Weight in this group was slightly above WHO standards, while height was lower. This was the group with the highest number of cases of students born pre-term (15%) and in which they practiced the least extracurricular physical activities (92.73% did not do so; Table 7).

**Table 7:** Comparison between real scores for weight and height in the study and those expected based on WHO Standard scores (2006).

		Real scores			Expected weight		Expected height		Expected BMI	
		Weight	Height	BMI	Girls	Boys	Girls	Boys	Girls	Boys
44 months	Cluster 4	16.03	99.86	16.01	15.34	15.68	100.31	101.04	15.29	15.40
48 months	Cluster 3	16.63	100.81	16.33	16.07	16.35	102.73	103.33	15.26	15.33
50 months	Cluster 2	17.69	105.26	15.82	16.43	16.68	103.90	104.45	15.25	15.30
58 months	Cluster 1	17.44	105.78	15.55	17.87	18.01	108.36	108.32	15.27	15.21

### 3.4 Relations of the four profiles to the biosocial variables

Finally, the relationships between each of the profiles described and some of the biosocial variables were analyzed (Table 8). No statistically significant differences were found concerning sex (*p* = 0.145) nor concerning prematurity (*p* = 0.066), but there were significant differences in terms of participation in extracurricular physical activities (*p* < 0.001).

**Table 8:** Chi-square test statistics regarding sex, prematurity, and extracurricular physical activities^a^.

		Cluster 1: high PMD (*n* = 121)	Cluster 2 high PMD except for left laterality (*n* = 129)	Cluster 3: medium-low PMD (*n* = 107)	Cluster 4 low PMD (*n* = 55)	Chi-square	V Cramer
*Sex*	Female	58 (47.93)	69 (53.49)	44 (41.12)	21 (38.18)	5.40	-
	Male	63 (52.07)	60 (46.51)	63 (58.88)	34 (61.82)		
*Prematurity*	No	114 (96.61)	90 (94.74)	85 (92.39)	34 (85.00)	7.18	-
	Yes	4 (3.39)	5 (5.26)	7 (7.61)	6 (15.00)		
*Extracurricular physical activities*	No	80 (66.12)	103 (79.85)	88 (82.24)	51 (92.73)	18.38**	0.21
	Yes	41 (33.88)	26 (20.15)	19 (17.76)	4 (7.27)		

*p < 0.05, **p < 0.001. ^a^The valid percentage is used, excluding missing values.

Although no overall differences were found for sex or prematurity, additional checks revealed statistically significant differences for prematurity between clusters 1 and 4 (Chi-Square = 6.79, *p* < 0.05) and clusters 2 and 4 (Chi-Square = 3.57, *p* < 0.05)—but the reliability of this result is scarce since the data used to calculate it is too small, as can be seen it (Table 8). Also, Table 9 shows statistically significant differences in participation in extracurricular physical activities in all clusters, except for 2 and 3.

**Table 9:** Chi-square test statistics for pairwise differences between profiles regarding extracurricular physical activities.

	Cluster	Chi-square test statistic	V Cramer
Extracurricular physical activities	Medium-low PMD (cluster 3) vs. High PMD (cluster 1).	7.62*	0.18
	High PMD, except left laterality (cluster 2) vs. Low PMD (cluster 4)	4.69*	0.16
	High PMD, except left laterality (cluster 2) vs. High PMD (cluster 1).	5.99*	0.16
	Low PMD (cluster 4) vs. High PMD (cluster 1).	14.07**	0.28

*p < 0.05, **p < 0.001.

In Table 10, the variance analysis showed differences between the groups by age between clusters 1 and the other three, with the oldest children being in cluster 1; there were also differences between clusters 2 and 4, with the average age being lower in cluster 4. As for the weight variable, there were differences between clusters 1 and 4, with the mean being higher in cluster 1 and, between clusters 2 and 4, higher in cluster 2. As for the height, differences between clusters 1 and, 3, and 4, and between 2 and 3 and 4 stood out, so that cluster 1 contains the tallest children, followed in order by clusters 2, 3, and 4.

**Table 10:** Analysis of variance for the variables age, weight, height, and BMI in the different profiles: ANOVA and *post-hoc* tests.

		Age			Weight			Height			BMI		
		Sum squares	*F*(3,408)	η^2^	Sum squares	F(3,408)	η^2^	Sum squares	F(3,408)	η^2^	Sum squares	F(3,408)	η^2^
		8699.4	32.54*	0.19	84.09	4.19*	0.04	1816.05	11.94**	0.10	29.23	4.91*	0.04
*Post-hoc*		M diff	SE	t	M diff	SE	t	M diff	SE	t	M diff	SE	t
C1	C2	7.08	1.20	5.93*	−3.66×10^−4^	0.35	−0.00	0.92	0.96	0.96	−0.28	0.19	−1.46
	C3	9.50	1.25	7.58**	0.81	0.38	2.11	4.96	1.05	4.71**	−0.79	0.21	−3.77**
	C4	13.35	1.54	8.70**	1.41	0.49	2.88*	5.92	1.34	4.41**	−0.47	0.27	−1.75
C2	C3	2.42	1.23	1.96	0.81	0.39	2.06	4.04	1.08	3.76**	−0.51	0.21	−2.39
	C4	6.27	1.52	4.12**	1.41	0.50	2.84*	5.00	1.36	3.67*	−0.19	0.27	−0.70
C3	C4	3.85	1.57	2.46	0.60	0.52	1.15	0.96	1.43	0.67	0.32	0.28	1.13

*p < 0.05, **p < 0.001. C1: High PMD, C2: High PMD (except left laterality), C3: Medium-low PMD, and C4: Low PMD.

## 4 Discussion

The study aimed to identify physical-motor profiles throughout preschool age (3–6 years) and explore associations between profiles and selected biosocial factors (age, sex, prematurity, weight, height, BMI, and participation in extracurricular physical activities).

Cluster analysis has made it possible to define four different groups of physical-motor development in childhood. These profiles have differed fundamentally in the level of development of laterality skills (right and left), coordination, balance, motor execution, and tonicity.

The type of groups that were formed showed that, although during physical-motor development, the child progressively acquires a set of differentiated skills, these must all be understood as part of a set, with development being compromised in some areas when the others are low. Proof of this is the fact that four profiles were obtained that differentiate between high overall skills (cluster 1) and high skills, except left laterality (cluster 2), medium-low skills (cluster 3), and low skills (cluster 4).

Regarding the difference between clusters 1 and 2, another of the findings of the present study is that, compared to other studies in which laterality is evaluated together, by separating the skills related to right laterality and the left, it has been found that the mastery of left laterality occurs in only one of the groups; specifically, in which it presents the greatest global development at a physical-motor level. The determination of the dominance of one or another member occurs gradually and depends on brain maturation, which can be influenced by aspects related to the environment and stimulation (Hill and Khanem, 2009; Betancourt et al., 2020). Around 90% of the world population are right-handers (Peters et al., 2006), and they tend to be more consistent in using their preferred side; that is, they tend to be more lateralized than a left-hander, who in turn is more symmetrical since he uses the non-preferred limb more frequently than right-handers (Gurd et al., 2006; Scharoun and Bryden, 2014; Bondi et al., 2020). Thus, left-handers seem to have more advantage in combined coordination tasks using both limbs (Freitas et al., 2014). Some studies have shown that ambidexterity is more common in athletes than in the general population (Stöckel and Weigelt, 2012; Stöckel and Vater, 2014) and that, in some cases, strong lateralization can be disadvantageous in the practice of certain physical activities those who require more open skills and whose use of the preferred and non-preferred hand is essential for their success (Nuri et al., 2013; Stöckel and Vater, 2014). For this reason, although we do not have data on the dominance of laterality in the sample, we can speculate that laterality is defined by age, brain maturation, the environmental context, and stimulus, so although the high levels of PMD the children in profile 2 are slightly younger than those in profile 1, these children also participate less in extracurricular physical activities and, as such, their left laterality may not be as improved as those in profile 1.

This study suggests that children with lower weights, especially those falling below the WHO weight standards (cluster 4), tend to have lower scores in physical-motor development when compared to children with higher development (cluster 1 and 2). An equivalent situation occurs, where boys and girls who are shorter than the WHO standards (clusters 3 and 4) also present less physical-motor development. These findings reinforce the idea that both weight and height can be important biological factors to consider when assessing and promoting the child’s physical motor development. Furthermore, it reinforces the assumption that motor development in children at very young ages is more influenced by biological maturation, and after this period, it becomes more influenced by environmental factors (stimuli/practice; Barnett et al., 2012).

Regarding extracurricular physical activities practice, the results are in line with many studies (Riethmuller et al., 2009; Holfelder and Schott, 2014; Valentini et al., 2016; Wick et al., 2017) since benefits could be deduced from this development by finding that those who practice extracurricular physical activities the most are in the high PMD group (cluster 1) and high PMD, except laterality (cluster 2).

Another element that we must consider is premature birth, although its relevance was only observed in the contrast between the high PMD group (cluster 1) and the low PMD group (cluster 4). Despite the lower number of premature children in this sample, these results are in line with several studies that indicate that premature children tend to have less motor competence than children who are born at full term (FitzGerald et al., 2021; Karageorgi et al., 2021).

While all these values may be relevant, there is no doubt that it is necessary to consider one of the critical variables in development: age. The data showed that children who have higher values of physical-motor development are also the oldest, being variable with significance in contrast between practically all groups [except the differences between high PMD (cluster 2) and medium-low PMD (cluster 3), as well as between this and low PMD (cluster 4)]. Motor skill development is a progressive process in which children typically acquire new skills and refine existing ones as they get older (Barnett et al., 2012; Valentini et al., 2016; Bolger et al., 2018; Li et al., 2023). It is also important to highlight that motor development depends on several biosocial factors, and, as such, not all children reach developmental milestones at the same time.

## 5 Conclusion, limitations, and future studies

These results lead us to question whether it is possible to establish profiles at such early ages. The answer we can approximate, based on the data, is considering the differences that exist in early development and the possible influence of multiple variables—not only biological but also environmental. This study allows us to identify specific trends that may be conducive to child development, such as participation in extracurricular physical activities. Other variables, such as sex, BMI, or prematurity, do not seem decisive when determining a higher or lower physical-motor development profile.

Given this data, analyzing these profiles in greater depth within broader age ranges and observing how they evolve could be interesting. This could provide a clearer idea of child development’s potential facilitators and risks. In this study, children’s postural status was not controlled. In future studies, it is recommended that this variable be incorporated, as well as other environmental variables that may be involved in these development profiles.

## Data availability statement

The raw data supporting the conclusions of this article will be made available by the authors, without undue reservation.

## Ethics statement

The studies involving humans were approved by Junta de Comunidades de Castilla-La Mancha. Ethical Committee of the CRA “Calar del Mundo.” The studies were conducted in accordance with the local legislation and institutional requirements. Written informed consent for participation in this study was provided by the participants’ legal guardians/next of kin. Written informed consent was obtained from the individual(s) for the publication of any potentially identifiable images or data included in this article.

## Author contributions

PG-M: Data curation, Investigation, Writing – original draft. LL-P: Formal analysis, Methodology, Writing – original draft. PM: Formal analysis, Methodology, Writing – original draft. CS: Conceptualization, Investigation, Supervision, Writing – review & editing. IS: Conceptualization, Supervision, Validation, Writing – review & editing. LS: Conceptualization, Supervision, Validation, Writing – review & editing.
